# Period tracker applications: What menstrual cycle information are they giving women?

**DOI:** 10.1177/17455065211049905

**Published:** 2021-10-09

**Authors:** Lauren Worsfold, Lorrae Marriott, Sarah Johnson, Joyce C Harper

**Affiliations:** 1 Institute for Women’s Health, University College London, London, UK; 2 Statistics and Data Management, SPD Development Company Ltd, Bedford, UK; 3 Clinical and Regulatory Affairs, SPD Development Company Ltd, Bedford, UK

**Keywords:** fertile window, menstrual cycle tracker, ovulation, period tracker app, periods

## Abstract

**Background:**

Period tracking applications (apps) allow women to track their menstrual cycles and receive a prediction for their period dates. The majority of apps also provide predictions of ovulation day and the fertile window. Research indicates apps are basing predictions on assuming women undergo a textbook 28-day cycle with ovulation occurring on day 14 and a fertile window between days 10 and 16.

**Objective:**

To determine how the information period tracker apps give women on their period dates, ovulation day and fertile window compares to expected results from big data.

**Methods:**

Five women’s profiles for 6 menstrual cycles were created and entered into 10 apps. Cycle length and ovulation day for the sixth cycle were Woman 1—Constant 28 day cycle length, ovulation day 16; Woman 2—Average 23 day cycle length, ovulation day 13; Woman 3—Average 28 day cycle length, ovulation day 17; Woman 4—Average 33 day cycle length, ovulation day 20; and Woman 5—Irregular, average 31 day cycle length, ovulation day 14.

**Results:**

The 10 period tracker apps examined gave conflicting information on period dates, ovulation day and the fertile window. For cycle length, the apps all predicted woman 1’s cycles correctly but for women 2–5, the apps predicted 0 to 8 days shorter or longer than expected. For day of ovulation, for women 1–4, of the 36 predictions, 3 (8%) were exactly correct, 9 predicted 1 day too early (25%) and 67% of predictions were 2–9 days early. For woman 5, most of the apps predicted a later day of ovulation.

**Conclusion:**

Period tracker apps should ensure they only give women accurate information, especially for the day of ovulation and the fertile window which can only be predicted if using a marker of ovulation, such as basal body temperature, ovulation sticks or cervical mucus.

## Introduction

Femtech includes applications (apps) that are dedicated to improving women’s health and addressing women’s health concerns. Femtech includes apps for fertility, pregnancy and menstrual cycle tracking and is estimated to be a US$50 billion industry within the next few years.^1,2^

Menstrual cycle or period tracking apps were first released in 2013.^1,2^ It is estimated that 50 million women worldwide use period tracker apps.^3^ The apps allow women to track their menstrual cycles and receive a prediction for the start of their future cycles. Almost all apps also provide predictions of the day of ovulation and the fertile window. Some of these apps have communicated that they use self-learning algorithms, declaring predictions are improved the longer a user tracks their data, which is primarily based on average cycles.^4,5^ Some required paid subscriptions to access the information. However, it is not known whether predictions match reality.

Our text book understanding of the menstrual cycle reports the average menstrual cycle length as being 28 days with ovulation on day 14. However, recent studies have expanded on the existing cohort data, publicizing a better understanding of regular cycle variability including length and ovulation day.^6^ Grieger and Norman’s^7^ retrospective global cohort study reported that out of 1.5 million users of the period tracker Flo, only 16% of women had a cycle median of 28 days with only 13.08% of cycles estimated to ovulate on day 14. Similar results were found by Bull et al., looking at over 600,000 cycles with data from the fertility and contraception app Natural Cycles. They showed only 13% of women had a 28-day cycle and these women had an average ovulation day of 15.4, increasing to day 19.5 for women with cycles of 31–35 days.^8^ Their study showed the average day of ovulation was day 16.9. Similarly, Johnson et al.^9^ found an average ovulation day of day 16 (21%; 14% for day 14). Data from over 75,000 users of a connected home ovulation test found day 15 was the most common ovulation day for a 28-day cycle (27%), with a 10-day spread of ovulation days and only 20% of women ovulating on day 14. Symul et al.^11^ analysed 2.7 million cycles and found that only 24% of ovulations occurred on day 14–15. There is considerable variation in cycle length both between and within women, with over half of women having cycles that vary by 5 or more days.^9^

There are markers of ovulation that can determine the ovulation day, such as basal body temperature (BBT), cervical mucus changes or the luteinizing hormone (LH) surge. But a study of 90 apps that are marketed as fertility apps found that 54% of apps only used calendar dates to predict ovulation rather than tracking BBT, cervical mucus or LH.^12^ Whether a woman is trying to get pregnant or not get pregnant, an app that tells her she is ovulating on a day decided simply by her cycle dates can be inaccurate. Li et al.^6^ demonstrated that tracking these other variables can be of use to clinicians and users for conditions such as endometriosis and polycystic ovaries, but highlights that it is difficult to know the true psychological experiences which vary between users.

The fertile window is considered to be 6 days in length, which includes the day of ovulation and the 5 days before.^13^ After ovulation, the egg is only viable for 24 h, but the fertile window includes the 5 days before ovulation as sperm can survive in the female genital tract for up to 5 days.^14^ The length of the fertile window exhibits intra- and inter-individual variation.^15,16^

To date, no studies have examined the period dates, ovulation day and fertile window predictions of period tracker apps. In this study, we have used real-life data for five ‘average’ women to determine whether 10 period tracker apps give women accurate information.

## Methods

### Selection of the apps

From January 2020 to May 2020, the Apple store and Google Play store were searched using ‘menstrual’, ‘menstruation’ or ‘period’ combined with either one or two additional terms from ‘tracker’, ‘cycle’, ‘calendar’, ‘predictor’ or ‘calculator’.

The 10 top reoccurring apps, with the most popular in descending order selected in the app stores, were decided following the inclusion and exclusion criteria. Inclusion: those marketed and used by individuals for tracking their menstrual cycle; apps with the ability to track cycles and input retrospective data giving predictions of the next menses, apps available in both the Apple and Google Play store, apps accessible in English and the ability to be used without an Internet connection. Exclusion: those that did not allow for the tracking of previous and present menstrual cycles or did not predict future menstrual cycles, apps whose sole purpose was for fertility, trying to conceive or pregnancy, apps not present in both Apple and Google Play stores, those not in English or after download those determined to be dysfunctional or faulty.

The chosen 10 period tracker apps were downloaded onto five devices on June 2020 (each containing all 10 apps) (Table 1). Each of the five devices was used solely for menstrual cycle data for one of the five women.

**Table 1.** The 10 apps used and the number of downloads.

App	Downloads May 2020
Clover Period Tracker Calendar	90k
Clue—Period & Cycle Tracker	400k
Femometer—Period & Fertility	90k
Flo My Health & Period Tracker	2M
Glow Period, Fertility Tracker	60k
Maya—My Period Tracker	<5k
Menstrual Calendar—Pinkbird	20k
My Calendar—Period Tracker	80k
Period Diary Pro	10k
Period Tracker—Eve	70k

The consistent data for each woman entered into the apps were 4 days of menses, aged 30, height and weight was decided from national averages of the UK female at 161 cm (5 ft 3″) and 71 kg (11 st 2 lbs).^17^

A virtual profile was created for each woman. Set up included emails and passwords for data backups, terms and conditions, and privacy policies accepted if applicable. App set-up was selected for ‘tracking your period’.

### Input of menstrual cycle data

The individual profiles for the five different women were built using thousands of cycles from a database of women using the Clearblue Connected Home Ovulation Test.^9^ Profiles were built to provide cycle lengths and ovulation dates for seven consecutive menstrual cycles, and these were transformed into dates to enable data entry into the apps (Table 2). The cycle length segments for each profile are shown in Table 3.

**Table 2.** The data underpinning the individual women’s profiles used to test the apps.

Woman	Average cycle length in days (type of cycle)	Number of cycles used to build profile	Number of women used to build profile	Days difference (shortest–longest cycle across six cycles)
1	28 (Constant)	N/A	N/A	±0
2	23 (Average)	1162	187	±3
3	28 (Average)	15,527	2113	±2
4	33 (Average)	6376	859	±4
5	31 (Irregular)	N/A	49	±6

**Table 3.** The cycle profiles of the five women that was inputted into the apps, and the expected ovulation day for cycle 6.

Woman	Cycle length (days)	Cycle length 1	Cycle length 2	Cycle length 3	Cycle length 4	Cycle length 5	Cycle length 6	Day of ovulation (Cycle 6)
1	28	28	28	28	28	28	28	16
2	23	22	23	25	21	22	24	13
3	28	27	29	26	28	30	29	17
4	33	36	32	33	31	29	33	20
5	31	31	39	30	27	34	26	14

Each of the five devices was consistently used for one woman only.

This study was set up as a matrix experiment, whereby 5 six-cycle profiles, representing the 90% percentile of biological range, were inputted into 10 tracking apps, representative of those commonly used by women. The array of cycles across a real-life population represents from the average short cycles on one end of the spectrum to long cycles on the other end, as well as including an intermediate average, the ‘textbook’ cycle and an irregular cycle on the same scale. Using Bull et al.’s^8^ research of 600,000 real-life cycles, and examining what percentage of cycles fall into these categories, ensures the five profiles used accurately represent a variety of real-life cycles. The actual data used to build the profiles was then taken from a second study.^18^ One difficulty to highlight was the creation of the cycle data for woman 5, as the irregular cycle data history, when averaged, decreased the irregularity. To combat this, it was decided to use fewer women’s cycle data, focusing on different cycle length individually. An advantage of using the Ovulation Test data base ensured objective and reliable data obtained from the original source.

Menstrual cycle data entry occurred in three phases, from 15 June 2020 to 22 July 2020: phase 1 consisted of the entry of the first four menstrual cycles; phase 2, correct entry of menstrual cycle 5 for each woman; and phase 3, final entry of cycle 6 at the correct time point, additionally adding the completion of menses.

The data collection occurred from 15 June 2020 to 28 July 2020 in three corresponding phases to data entry. In phase 1, menstrual cycle predictions/estimation dates were noted along with the fertile window and day of ovulation if applicable for cycles 5, 6 and seven. Phases 2 and three followed the same data collection as set out in phase 1 for cycles 6 and 7.

Upon completion of entering and collecting predicted menstrual cycle dates, cycle length was calculated for each and compared to real-life menstrual cycle length data with the difference calculated. In phase 1, difference was calculated between predicted cycle length for cycles 5 and 6 and the corresponding actual cycle length. In phase 2, cycle length difference was calculated for cycles 5 and 6 with correct start date for menses five. In phase 3, cycle length difference was calculated between cycle 6 with the correct start date and cycle 7. This was undertaken for each individual woman (1–5) and for each of the 10 apps used, in every phase.

Further analysis of cycle length was undertaken looking at how cycle length changed between the addition of retrospective and current menses dates on each app classified for each woman. This allowed for the discovery of how menses predictions changed cycle length over time for an individual woman on a specific app.

Fertile windows and day of ovulation, if available, from each women and app were converted from a date to the day within the cycle, from each phase of data collection. This allowed tracking of whether the day was static or altered between women’s varying cycle length within apps.

### Ethics

Ethical approval was not sought because no human volunteers were involved in the study. Data used to determine profiles were from already published sources. This study of menstrual cycle tracking apps using menstrual cycle lengths and including evaluation of the used apps did not directly involve or pose any risk to human subjects and consequently did not require consent from an ethical review board. All users of Clearblue ConnectedHome Ovulation Test provide consent for their data to be used for research purposes and results to be published in an anonymized fashion.

## Results

For each cycle that was entered into the apps, a number of outcomes were obtained including predicted start of the next period, predicted ovulation day and predicted fertile window. These data were collected for each of the six cycles. The results presented below focus on cycle 6 results which gave each app the previous cycles to learn from.

### Menstrual cycle lengths

**Figure 1.**
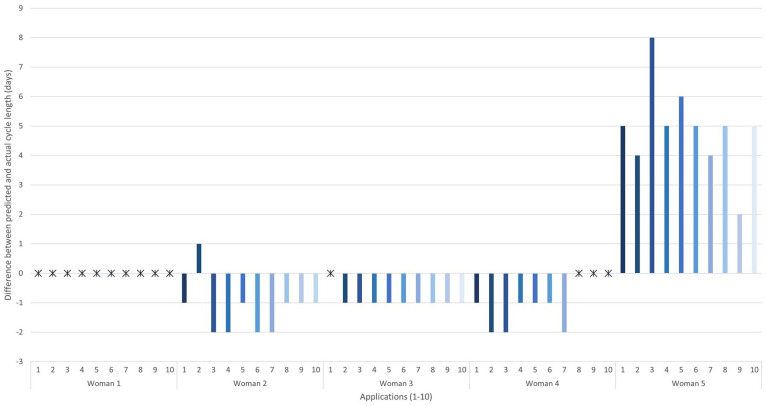
The difference between predicted and actual cycle length, in days, for cycle 6. The asterisk denotes a correct prediction. Columns below the line were predicted shorter than expected and those above the line were longer than expected.

For cycle length, the apps all predicted woman 1’s cycles correctly. Prediction of the exact day of menses was rare for women 2–4, with apps generally predicting the period to arrive 1–2 days earlier than the true day. Prediction for woman 5 (irregular cycles) ranged from 2 to 8 days longer than the true day of menses (Figure 1).

**Figure 2.**
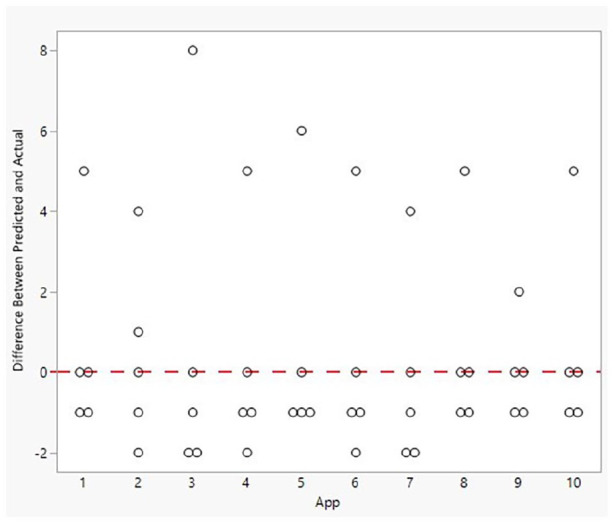
Difference between each woman’s predicted and actual cycle length from each App.

Assessing cycle length across all five women for each app, the least accurate was app 3 ranging from −2 to 8 days from the actual cycle length for all women (Figure 2). App 9 was the most accurate ranging from −1 to 2 days from the actual cycle length.

### Ovulation

**Figure 3.**
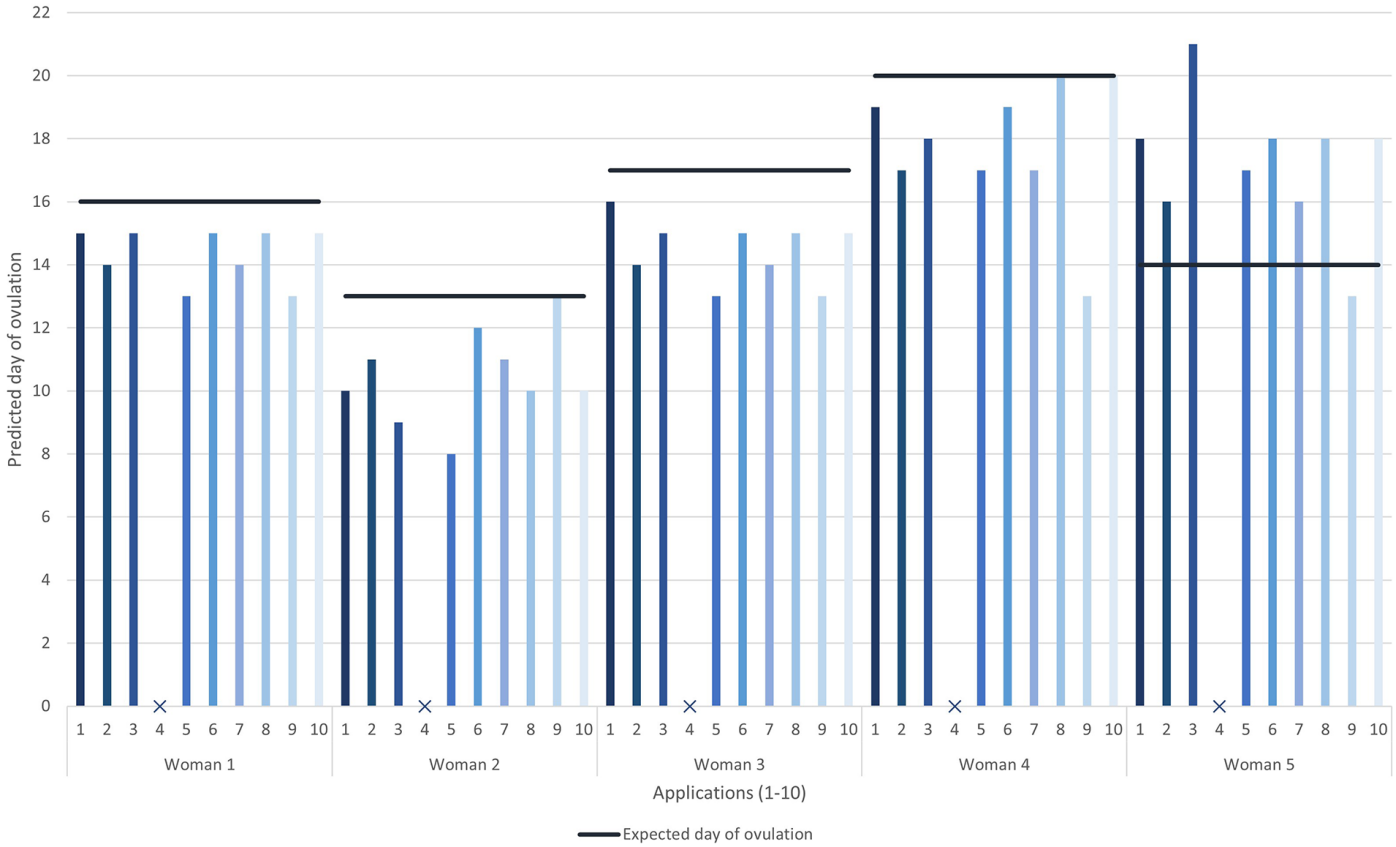
The predicted ovulation day for cycle 6 for each woman, by each app. The solid horizontal line is the expected ovulation day.

The predicted ovulation day for cycle 6 for each woman, by each app is shown in Figure 3. App 4 did not provide a predicted ovulation day. For women 1–4, of the 36 predictions, 3 (8%) were exactly correct, 9 predicted 1 day too early (25%) and 67% of predictions were 2–9 days early. For woman 5, most of the apps predicted a later day of ovulation (−1 to 7 days range of predictions).

### Fertile window

**Figure 4.**
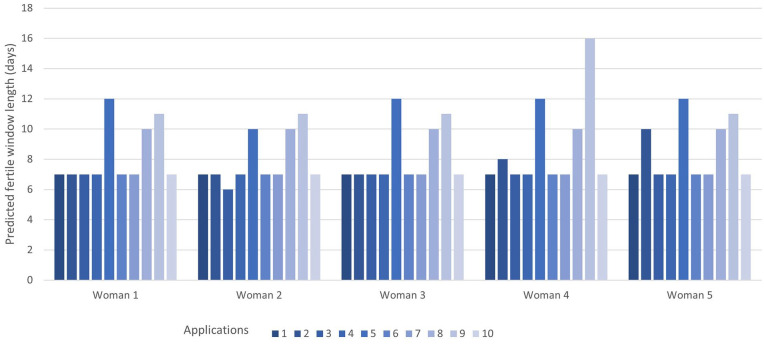
The predicted fertile window length for cycle 6 for each woman by each app.

All apps provided a predicted fertile window length for cycle 6 (Figure 4). Apps 1, 4, 6, 7 and 10 predicted a 7-day fertile window in every case, irrespective of cycle length.

App 2 predicted a 7-day fertile window in all cases except women 4 and 5, where it predicted an 8- and 10-day fertile window, respectively. App 3 predicted a 7-day fertile window in all cases except woman 2, where it predicted a 6-day fertile window. App 5 predicted a 12-day fertile window in all cases except woman 2 where it predicted a 10-day window. App 8 predicted a 10-day fertile window for all women. App 9 predicted an 11-day fertile window except for woman 4 where it predicted a 16-day window.

## Discussion

The aims of this study were to determine whether 10 period tracker apps gave accurate predictions of cycle length, ovulation day and the fertile window for five ‘average’ women. The study design where results were generated from the range of possible cycle characteristics was important, as while the Apps perform similarly for the average data, it was not possible to predict how they would perform for more varied cycles. We found that the period trackers gave conflicting information for cycle and fertility prediction.

The five women’s cycle profiles were chosen to represent a wide range of real-life women, including a ‘metronome’ 28-day cycle, profiles for women who on average have short, average or long cycles and a woman with irregular cycles, thus testing the apps abilities to predict menstrual cycles across a range of cycle types (Table 3). We predicted that the apps would deal very well with the ‘metronome’ cycle but have the most issues with the irregular cycle. In the sample analysed by Bull et al.,^8^ 13% of women had a regular 28-day cycle similar to woman 1, 8% of women had a cycle similar to woman 2, 65% to woman 3 and 19% to woman 4. Women with very irregular cycles ranging from 36 to 50 days made up 7% of women.

### Cycle length

As expected, all the apps predicted woman 1’s 28-day cycle correctly. But for the other women, after six cycles, the apps rarely got the next cycle prediction correct. For women 2, 3 and 4, the majority of apps predicted a shorter cycle than expected and for woman 5 they predicted a longer cycle.

Our advice for women is that the predicted next period date should not be relied on by any woman who does not have a regular 28-day cycle and the apps could be out by as much as 8 days. As long as women understand that their period may arrive earlier or later than predicted, and do not get concerned about it, we do not see this as a negative aspect of the apps. It may be beneficial for apps to provide women some understanding of their likely accuracy for period prediction.

There is value in the historical data that period tracker apps store. By having a record of their menstrual cycle history, women may be alerted to changes which may affect their reproductive health. Women may keep a record of premenstrual syndrome symptoms and have some idea of when to expect this. If they are having irregular cycles or cycles shorter than 21 days or longer than 35 days, they may wish to see a health professional, especially if they are trying to get pregnant. Women who are peri-menopausal may find it useful to have an accurate record of how their cycles are changing and when their last period was. For women trying to get pregnant, having an accurate record of their last menstrual period helps to date the pregnancy. All of this information will be valuable if they need to see a health professional about their reproductive health.

### Day of ovulation

For women with regular 28-day cycles, most women will ovulate on day 15 or 16.^8,10,18^ Besides woman 5, all apps that predicted ovulation day predicted it earlier than we expected. Even for women 1 and 3 with 28-day cycles, the majority of apps predicted ovulation as day 14 or 15. Predicting ovulation day earlier than expected is particularly problematic if women are having unprotected sex when they think they are 24 h past ovulation. They may think their fertile window is closed, but they may not have ovulated and will be at high risk of pregnancy. And the converse is that for woman 5, ovulation occurs earlier than predicted by the apps so if she is planning a pregnancy, she may miss her fertile window.

Ovulation day can only be determined if a marker of ovulation is measured, such as BBT, cervical mucus or LH surge.^12^ Period tracker apps that solely look at period dates should not be providing women with incorrect information about their possible ovulation day. App 4 was the only app that did not give a predicted day of ovulation; however, it did give a prediction of the fertile window.

### Fertile window

Most of the 10 apps predicted the fertile window as 7 days for all women. Those apps predicting a longer fertile window length generally gave this window consistently for each woman.

It is also important to consider that the fertile window is not a fixed length for all women or all cycles. It begins when oestrogen rises, changing the consistency of the cervical mucus to become supportive for sperm survival. In some cycles, the oestrogen rise occurs many days before ovulation, resulting in a long fertile window, while in others oestrogen rise can occur on the day of the LH surge.^19,20^

When menstrual cycle data is used to predict the fertile period for natural family planning contraception (calendar method), the fertile window predicted is generally very long to prevent women being at high risk of an unwanted pregnancy. These methods also indicate their low contraceptive reliability. There is a risk when providing a 7-day fertile window to women without emphasizing that it should not be used for contraceptive purposes as women could end up with an unwanted pregnancy due to incorrect information. Similarly, a woman seeking to conceive may mistime intercourse, with the risk of delaying her time to pregnancy.

Levy and Romo-Avilés^21^ performed 26 qualitative interviews to ask women about their experience using period tracker apps. The women found the apps empowering and they gave the women the opportunity to gain knowledge and be more aware of their menstrual cycles. They could record any menstrual cycle irregularities and use this information for health professionals. But some anxiety was reported including concern when their period came at a date different to that predicted, which our study shows would be a regular occurrence. Zwingerman et al.^22^ reviewed menstrual cycle apps and concluded that many were of poor quality, reporting that 22.1% contained serious inaccuracies in content, tools or both. An audit of period tracker apps reported that most apps were not evidence based^23^ which our study shows is especially true for ovulation day and the fertile window predictions.

## Limitations

The main limitation of this study is that we only inputted 6 months of cycle data and over time with increased data input apps should learn to accurately predict somewhat regular cycles. However, it is unclear whether this would be the case. In theory this should happen for ‘regular’ cycles, but, not for irregular cycles, which by definition are not predictable so would not benefit from expanded data input. But either way, apps should not be giving women information about their ovulation and fertile window when solely looking at cycle dates,^12^ at least not without making it very clear that the predictions are ‘guestimates’ that should not be used for family planning purposes. This study only considered the top 10 Apps meeting the study criteria, so some less used Apps could have superior performance criteria to those most downloaded from the App store. The Apps were downloaded in the United Kingdom, and it is possible that the same App downloaded from a store outside of the United Kingdom may also have different performance characteristics. The study design took the approach of considering profiles of average and diverse women, but there are obviously countless permutations in between that have not been tested. A simulation using a large-scale dataset of real women’s profiles would offer the most accurate view of performance; however, the sheer scale of data entry make this impracticable.

## Conclusion

For women, cycle length prediction can be useful to have an idea of when their period might arrive and the possible onset of premenstrual syndrome (PMS). The value of having an accurate record of their menstrual cycles may be very important for some women, for their fertility, pregnancy and during the perimenopause.

Our recommendation to app companies is to stop giving women inaccurate predictions for ovulation and the fertile window; they should include monitoring a marker of ovulation to provide these predictions.

Education about women’s reproductive health is lacking in all countries. In the United Kingdom, in 2019 for the first time the Department for Education included the need for education into ‘the facts about reproductive health, including fertility and the potential impact of lifestyle on fertility for women and women and menopause’ in their Relationships Education, Relationships and Sex Education (RSE) and Health Education Curriculum.^24^ With many women using period tracker apps, it gives the ideal opportunity for women to have a platform to learn about their menstrual cycle health.
